# Synovial fluid calprotectin in diagnosing periprosthetic joint infection: A meta-analysis

**DOI:** 10.1007/s00264-022-05357-6

**Published:** 2022-03-02

**Authors:** Ashraf T. Hantouly, Motasem Salameh, Ahmad A. Toubasi, Loay A. Salman, Osama Alzobi, Abdulaziz F. Ahmed, Shamsi Hameed, Bashir Zikria, Ghalib Ahmed

**Affiliations:** 1 Department of Orthopedic Surgery, Hamad Medical Corporation, Doha, Qatar; 2 Department of Orthopaedic Surgery, The Warren Alpert Medical School at Brown University, 1 Kettle Point Avenue, East Providence, RI 02915 USA; 3 Faculty of Medicine, University of Jordan, Amman, Jordan; 4 Department of Orthopaedic Surgery, Johns Hopkins School of Medicine, Baltimore, MD USA; 5 Aspetar Orthopaedic and Sports Medicine Hospital, Doha, Qatar; 6 Department of Orthopaedic Surgery, Surgical Specialty Center, Hamad Medical Corporation, Doha, Qatar

**Keywords:** Periprosthetic joint infection, Arthroplasty, Diagnosis, Calprotectin, Diagnosis, Synovial

## Abstract

**Purpose:**

Periprosthetic joint infection (PJI) is one of the most debilitating complications following joint replacement surgery. Synovial biomarkers, such as Calprotectin, have become valuable in the diagnosis of PJI. This meta-analysis aimed to investigate the role of synovial Calprotectin as a diagnostic test in PJI.

**Methods:**

This meta-analysis was conducted with adherence to PRISMA guidelines. PubMed, Cochrane, Web of Science, and Google Scholar were searched until February 2022. Inclusion criteria were as follows: all studies in which the patients with joint replacements were evaluated for PJI; synovial Calprotectin was the biomarker of choice to diagnose PJI; standardized guidelines were used as the gold standard for the diagnosis; and a comparison between the guidelines and Calprotectin results was made. Diagnostic parameters such as sensitivity, specificity, diagnostic odds ratio (DOR), positive predictive value, negative predictive value, and area under the curve (AUC) were calculated for the included studies to evaluate synovial Calprotectin for PJI diagnosis.

**Results:**

The total number of the included patients was 618 from eight studies. The pooled sensitivity, specificity, and diagnostic odds ratio of Calprotectin test were 92% (95%CI: 84%-98%), 93% (95%CI: 84%-99%), and 187.61 (95%CI: 20.21–1741.18), respectively. The results showed that the negative and positive likelihood ratios of the Calprotectin test were 0.07 (95%CI: 0.02–0.22) and 9.91 (95%CI: 4.11–23.93), respectively. The SROC showed that the area under the curve for Calprotectin test was 0.935.

**Conclusion:**

Synovial Calprotectin is a valuable biomarker as it provides a reliable and rapid diagnosis of PJI. It has the potential to be used in clinical practice due to its high sensitivity and specificity that are comparable to the other utilized biomarkers. Another advantage is its low cost relative to other biomarkers.

## Introduction

Periprosthetic joint infection (PJI) is defined as an infection of prosthesis and the surrounding soft tissues and is considered one of the most debilitating complications following joint replacementsurgery. PJI contributes to around 14% of all knee and hip revision arthroplasties [1], leading to an enormous healthcare and economic burden that adds up to $1.62 billion in the USA alone [2]. However, the accurate and timely diagnosis of PJI remains quite challenging with wide variation based on the standard adopted guidelines.

Efforts to standardize the diagnosis of PJI yielded various guidelines of pre-operative and intra-operative criteria by the Musculoskeletal Infection Society (MSIS) and International Consensus Meetings (ICMs) [3–5]. In 2018, a new validated and updated version of the MSIS criteria was defined by Parvizi et al. with a higher sensitivity of 97.7% compared to the original MSIS (79.3%) and ICM definition (86.9%), with a similar specificity of 99.5% [4]. However, the inclusion of microbial cultures in these criteria remains a setback due to their poor reliability (sensitivity and specificity), particularly with low-grade micro-organism infections [6].

While serologic markers such as CRP, D-dimer, ESR have been widely used in the diagnosis of PJI, they are highly influenced by various systemic and confounding factors [7, 8]. The emergence of new diagnostic modalities has made synovial biomarkers of particular interest, including synovial WBC, leukocyte esterase, Alpha-Defensin, and Calprotectin, which have shown promising potential as diagnostic tools in PJI.

Calprotectin, also known as cystic fibrosis antigen, is a protein complex mainly secreted by neutrophils as part of the inflammatory response and plays a role in leukocyte migration and stimulation [9]. Different testing methods have also been explored to detect synovial Calprotectin, including enzyme-linked immunosorbent assay (ELISA) and lateral flow testing, showing promising results [10, 11]. Several studies have reported the efficacy of synovial Calprotectin in the diagnosis of PJI; however, further understanding of the underlying pathophysiology and diagnostic accuracy is warranted. Therefore, high-quality evidence is needed to highlight the reliability of synovial Calprotectin as a diagnostic tool in PJI.

This meta-analysis aimed to investigate the role of synovial Calprotectin as a diagnostic test in PJI and measure its reliability and validity in terms of sensitivity, specificity, diagnostic odds ratio (DOR), positive predictive value, negative predictive value, and area under the curve (AUC).

## Materials and methods

This systematic review and meta-analysis were conducted with strict adherence to the Preferred Reporting Items for Systematic Reviews and Meta-Analyses (PRISMA) guidelines [12]. The focus was studies that compared Calprotectin, as a biomarker to diagnose PJI, with gold standard criteria such as the MSIS and ICM-2018.

### Information sources and search strategy

Electronic databases of PubMed, Cochrane, Web of Science, and Google Scholar were searched from inception till February 2022. The following keywords were used: “Periprosthetic joint infection” OR “Prosthesis-related infections” AND “Synovial” AND “Calprotectin.” Two independent reviewers screened the titles and abstracts, and the full-text review was done for the eligible studies as per the below-mentioned criteria.

### Eligibility criteria

All articles were included if the following criteria were met:

- Patients with joint replacements being evaluated for PJI.
- Synovial fluid aspiration was done for PJI diagnosis.
- Standardized diagnostic criteria, such as MSIS and ICM-2018, were used to diagnose PJI.
- Calprotectin was used as a biomarker to diagnose PJI.
- A comparison between Calprotectin and the diagnostic criteria was done.
- We only included accessible articles that were published in English.

### Exclusion criteria

Studies that did not use standardized criteria were excluded. We also excluded studies that did not use Calprotectin among the biomarkers for PJI diagnoses. Patients who had a first-stage revision before being investigated for PJI with Calprotecin were excluded.

### Data collection process and data items

We collected the following data items: Author’s name, study year, country of origin, age, sex, number of participants, diagnostic criteria, detection method, Calprotectin cutoff point, Calprotectin sensitivity, Calprotectin specificity, Calprotectin positive predictive value, Calprotectin negative predictive value, Area Under the Curve, Calprotectin concentration in septic and aseptic joints.

### Risk of bias in individual studies

Two of the authors evaluated the methodological quality of the included studies using the QUADAS-2 tool, which is composed of four key domains; patient selection, index test, reference standard, and flow and timing [13]. Signaling questions were applied to evaluate the risk of bias and clinical applicability. The risk of bias is judged as “low,” “high,” or “unclear” (when insufficient data are reported to permit a judgment). Any disagreement between the two authors was resolved by a discussion with a senior author.

### Statistical analysis

For all the studies, we constructed a 2 × 2 contingency table, then the sensitivity, specificity, diagnostic odds ratio, positive and negative predictive values were calculated for each study. Moreover, we pooled the prevalence of the disease in the included studies using a random effect model with double arcsine transformation to calculate the diagnostic parameters that need prevalence to be calculated (PPV and NPV). When more than one threshold was used by any of the included studies, the threshold with the largest Yourdon index was used in the analysis. The mentioned diagnostic parameters were pooled using a random effect model. In addition, the summarized receiver operating characteristic (SROC) curve was constructed using these diagnostic parameters. The heterogeneity of the included studies was investigated using the Cochrane Q and I2 statistic. All the mentioned analyses except the SROC were conducted using Meta XL, version 5.3 (EpiGear International, Queensland, Australia). The SROC was generated using MetaDTA: Diagnostic Test Accuracy Meta-Analysis v2.01 [14].

## Results

### Study Selection

**Fig. 1:**
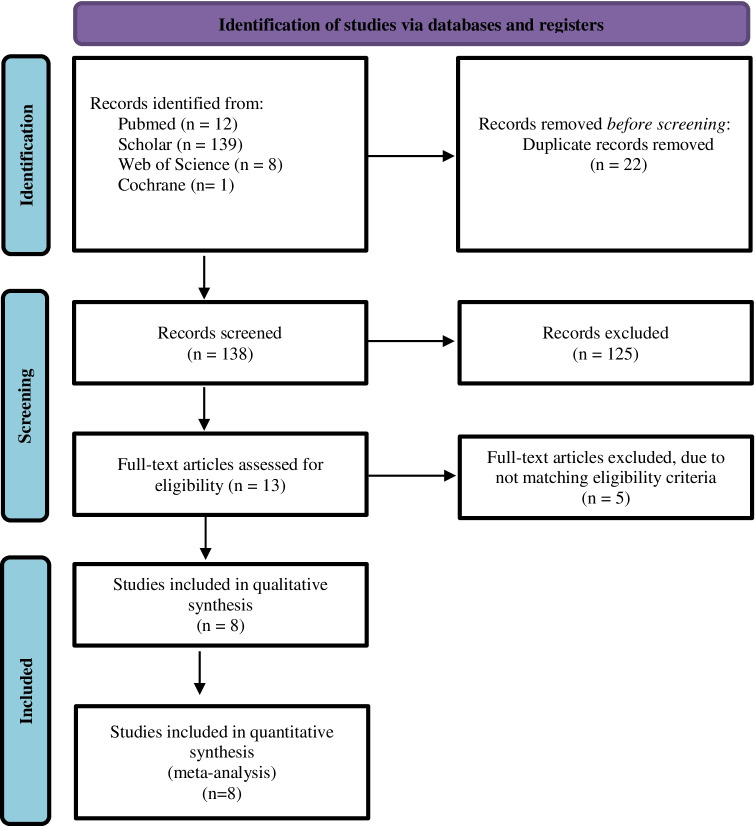
Search strategy flowchart

The search yielded 160 articles, 22 of which were duplicates that were removed manually and electronically. After screening using title/abstract, 125 were excluded. The remaining 13 articles were screened using a full-text form, and five of them were excluded. Finally, eight articles were included in this study. The detailed selection process is described in Fig. 1.

### Characteristics of the included studies

A total number of 618 patients from eight studies were included in this meta-analysis. The percentage of septic patients in the included studies according to the ICM or MSIS criteria was 39.5% (244/618) while 60.5% of them were aseptic patients (374/618). Four studies specified the number of septic and aseptic patients according to gender using the ICM/MSIS criteria. Among the male patients in these studies, 19.7% were septic (55/127), while 40.7% of the female patients were septic (83/204) Two of the included studies used ELISA as a detection technique, two studies used point of care and ELISA tests, three studies used lateral flow immunoassay, and one study used immunoturbidimetric Calprotectin immunoassay. Salari et al. [15], WouthuyzenBakker et al. 2017 [16], WouthuyzenBakker et al. 2018a [17] and Grassi et al. [18] used the value of more than or equal 50 mg/l as their cutoff point for positive tests. On the other hand, the cutoff point for positive tests varied between Trotter et al. [10], Zhan et al. [11], and Grzelecki et al. [19], which were >  = 14 mg/l, > 173 ug/ml, and >  = 1.5 mg/l, respectively. In addition, Warren et al. [20] used two cutoff points for positive Calprotectin tests which were more than or equal 50 mg/l and more than or equal 14 mg/l. The more than or equal 50 mg/l showed higher Youden index value; hence, it was the one that was used in the analysis. Grassi et al. reported all parameters for both ELISA and POC test. However, the POC test they used was a protoyle, and therefore, the parameter calculated for ELISA test was included in the analysis. The characteristics of the included studies are described in Table 1.

**Table 1:** Studies characteristics

Study	Country	Study design	Participants (M/F)	Age	Detection method	Gold standard	Cutoff point	Septic Joints	Aseptic Joints	S*	SP*	AUC	PLR	NLR	PPV	NPV	Calprotectin concentration in septic Vs. aseptic joints
Salari, 2019 **✚**	Italy	Cohort	72 (36/40)	69	ELISA	ICM 2018	> = 50 mg/L	Knee 28	Knee 44	100%	95%	0.996	22	0	-	-	320 mg/L Vs 5.5 mg/L
Warren, 2021	USA	Cohort	123 (57/66)	Septic 66.9 ± 10.6 Aseptic 65.4 ± 10.6	POC + ELISA	MSIS	^ > = 50 mg/L > = 14 mg/L	Knee 53	Knee 70	98.1%	95.7%	0.969	-	-	94.50%	98.50%	-
Wouthuyzn-Bakker, 2017	Netherl-ands	Pilot	61 (25/36)	Septic 65 (24–87) Aseptic 60 (23–90)	Lateral Flow Immunoassay	MSIS	> = 50 mg/L	Knee 5 Hip 11 Shoulder 3	Knee 5 Hip 34 Shoulder 2 Ankle 1	89%	90%	0.94	8.9	0.1	81%	95%	991 mg/L Vs 11 mg/L
Wouthuyzn-Bakker, 2018	Netherl-ands	Cohort	52	-	Lateral Flow Immunoassay	MSIS	> = 50 mg/L	Knee 5 Hip 8 Shoulder 2	Knee 12 Hip 24 Shoulder 1	86.7%	91.7%	0.94	10.9	0.14	81.3%	94.4%	859 mg/L Vs 7 mg/L
Zhng, 2020	China	Cohort	63 (20/43)	Septic 64 (54–83) Aseptic 57 (41–86)	ELISA	MSIS	173 μg/ml	Knee 6 Hip 15	Knee 12 Hip 30	92.2%	976%	0.993	39.6	0.049	95.2%	97.6%	776 μg/m Vs 54.5 μg/m
Trotter, 2020 #	UK	Pilot	69 (37/32)	74.3 (45–89)	Lateral Flow Immunoassay	ICM 2018	> = 14 mg/L	Knee 9 Hip 15	Knee 8 Hip 37	75%	75.56%	0.78	-	-	62.07%	85%	-
Grzelecki, 2021	Poland	Cohort	85 (25/60)	Septic 65.5 ± 10 Aseptic 68.3 ± 12	Immunoturbidimetric Calprotectin Immunoassay	ICM 2018	1.5 mg/L	Knee 25 Hip 20	Knee 25 Hip 15	95.6%	95%	-	-	-	95.50%	95.00%	20.4 mg/L Vs 0.7 mg/L
	Italy	Cohort	93 (42/51)	**77**	ELISA + POC	ICM 2018	> = 50 mg/L	Knee 39	Knee 50	ELISA 92.3% POC 97.4%	ELISA 100% POC 94%	ELISA 0.962 POC 0.957	ELISA - POC 16.239	ELISA 0.077 POC 0.027	ELISA 100% POC 92.7%	ELISA 94.3% POC 97.9%	ELISA 290.6 mg/L Vs 6.5 mg/ L

**✚** 4 patients were excluded from the analysis due to inconclusive results and not all minor criteria were considered for all patients

^#^ Minor criteria of ICM 2018 were not considered

S*: Sensitivity

SP*: Specificity

PLR: Positive likelihood ratio

NLR: Negative likelihood ratio

^ Two cutoff points were used, the >  = 50 mg/L was used in the analysis

POC used in the study is a prototype. Thus, ELIZA parameters were used in the analysis

### Quality Assessment

**Fig. 2:**
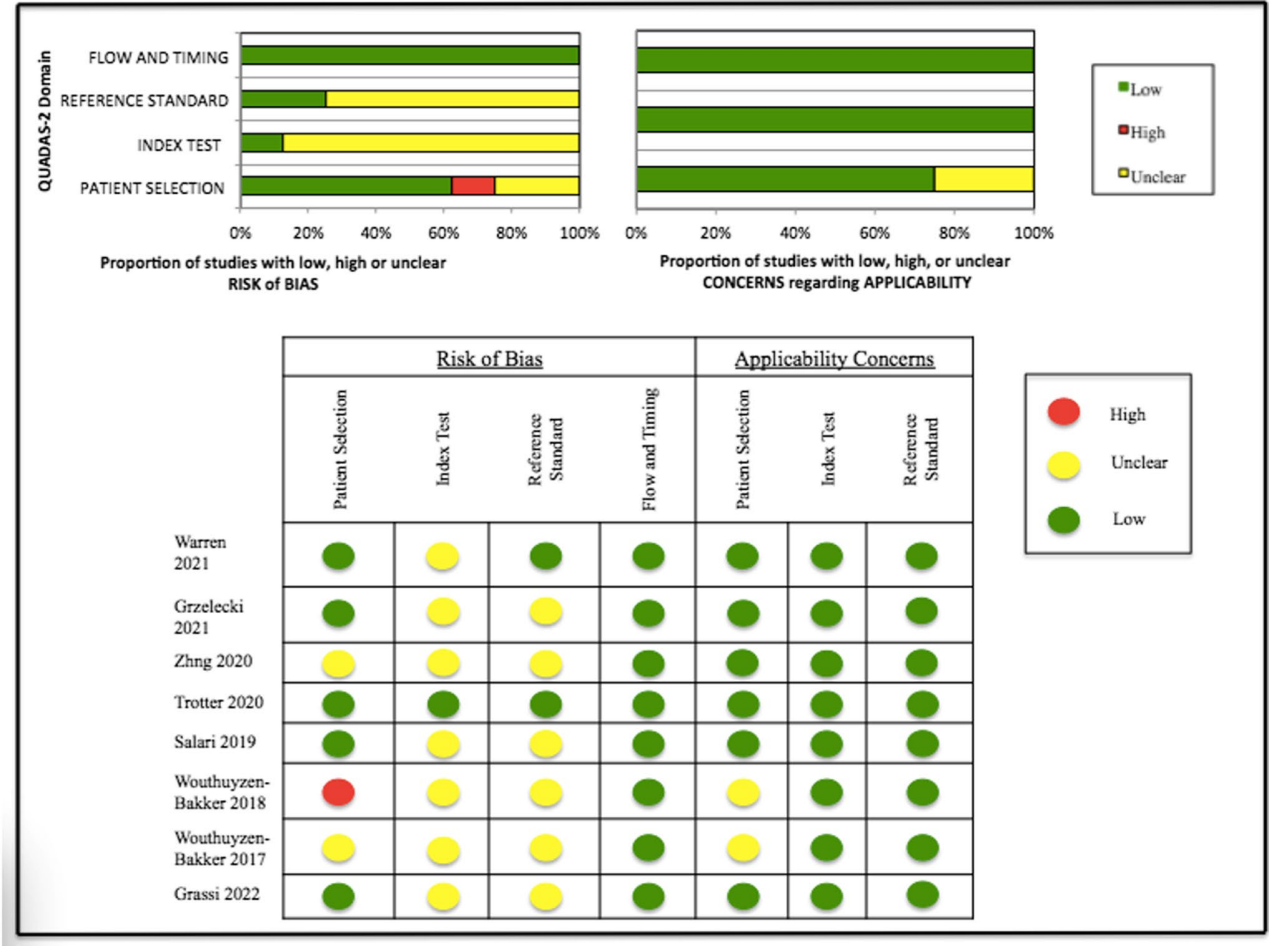
Quality assessment of the included studies using QUADAS-2 tool criteria

Figure 2 illustrates the quality assessment of the included studies using QUADAS-2 tool criteria.

### Sensitivity

**Fig. 3:**
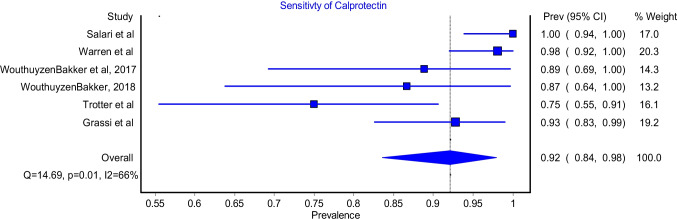
Sensitivity for Calprotectin test

The Calprotectin test sensitivity model included six studies. The model showed that the Calprotectin test had a sensitivity of 92% (Fig. 3; 95%CI: 84%-98%). The model had significant heterogeneity (Fig. 3; I^2^ = 66%, P-value = 0.01). The highest sensitivity was reported by Salari et al., and it was 99.6%, whereas the lowest sensitivity was reported by Trotter et al. and it was 75.0%.

### Specificity

**Fig. 4:**
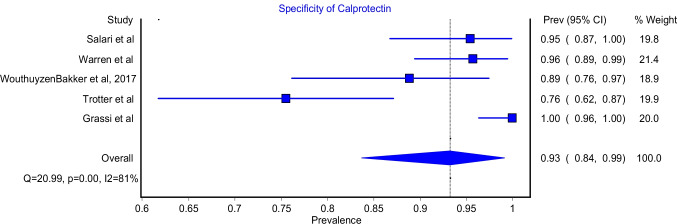
Specificity for Calprotectin test

The specificity of the Calprotectin test model included five studies. The model showed that the Calprotectin test had a specificity of 93% (Fig. 4; 95%CI: 84%-99%). The model had significant heterogeneity (Fig. 4; I^2^ = 81%, P-value = 0.00). The highest specificity was reported by Grassi et al. who reported a 100%% specificity for the test. On the other hand, the lowest specificity was reported by Trotter et al. and it was 75.6%.

### Positive and negative likelihood ratio

**Fig. 5:**
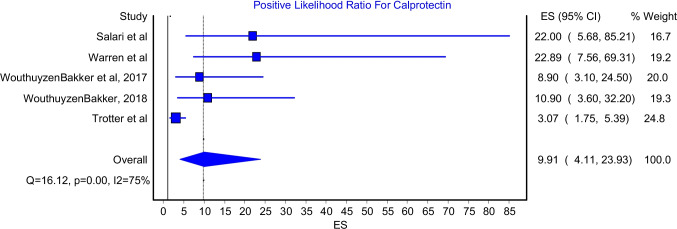
Positive likelihood ratio for Calprotectin test

**Fig. 6:**
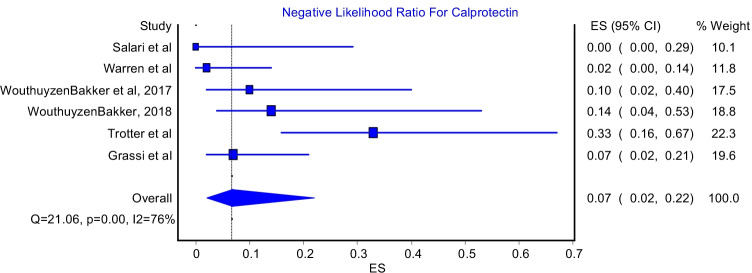
Negative likelihood ratio for Calprotectin test

The positive likelihood ratio model included five studies while the negative likelihood ratio model included six studies. The pooled positive likelihood ratio was 9.91 (Fig. 5; 95%CI: 4.11–23.93). This model had significant heterogeneity (Fig. 5; I^2^ = 75%, P-value = 0.00). The highest positive likelihood ratio was reported by Zhang et al. who reported a value of 39.6, while the lowest positive likelihood ratio was reported by Trotter et al. and it was 3.07. Moreover, the pooled negative likelihood ratio was 0.07 (Fig. 6; 95%CI: 0.02–0.22). This model had significant heterogeneity (Fig. 6; I^2^ = 76%, P-value = 0.00). The lowest negative likelihood ratio was reported by Salari et al. who reported a value of 0. In contrast, the highest negative likelihood ratio was reported by Trotter et al. which was 33.0.

### Diagnostic Odds Ratio

**Fig. 7:**
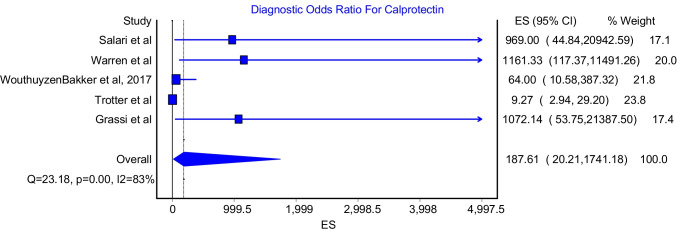
Diagnostic odds ratio for Calprotectin test

The diagnostic odds ratio model for the Calprotectin test included five studies. The pooled diagnostic odds ratio was 187.61 (Fig. 7; 95%CI: 20.21–1741.18). This model showed significant heterogeneity (Fig. 7; I^2^ = 83%, P-value = 0.00). The highest diagnostic odds ratio was reported by Warren et al. and Grassi et al., which were 1161.33 and 1072.14, respectively, whereas the lowest diagnostic odds ratio was reported by Trotter et al. which was 9.27.

## The Summary of Receiver Operating Characteristics Curve

**Fig. 8:**
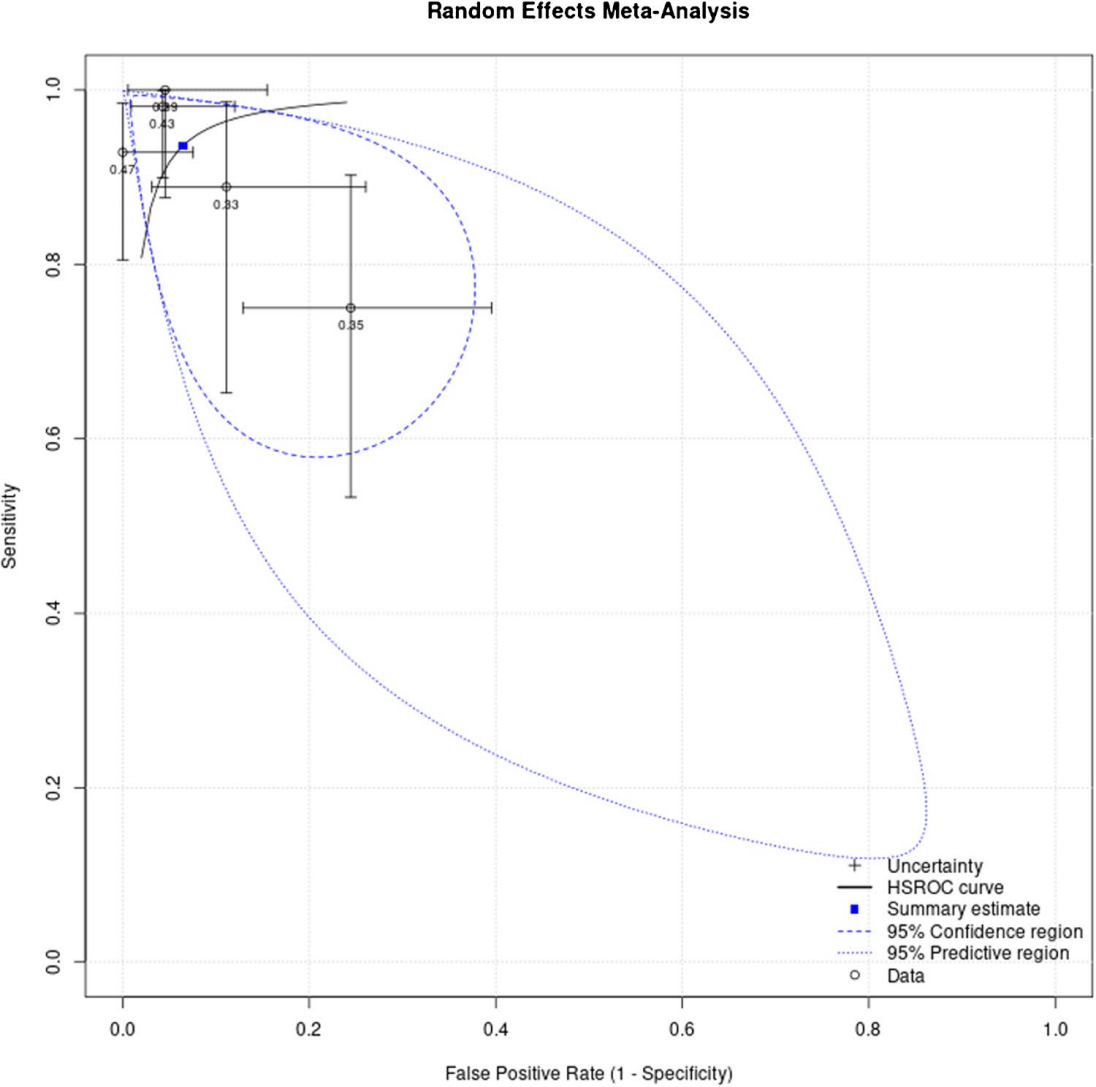
Meta-analysis for summary of ROC

The Summary of Receiver Operating Characteristics Curve (SROC) model for the Calprotectin test included five studies (Table 2). The SROC showed that the area under the curve (AUC) for the Calprotectin test was 0.935 (Fig. 8). The highest AUC was reported by Salari et al. which was 0.996 while the lowest was reported by Trotter et al. at 0.78. Moreover, the pooling of the studies that were included in the SROC revealed that the sensitivity and specificity for the Calprotectin test were 93.6% (95%CI: 83.5%-97.7%) and 93.5% (95%CI: 84.5%-97.5%), respectively. The positive and negative likelihood ratio for the Calprotectin test in the studies that were included in the SROC was 14.469 (95%CI: 5.571–37.579) and 0.068 (95%CI: 0.024–0.192), respectively. Additionally, the diagnostic odds ratio for the Calprotectin test in the studies that were included in the SROC was 212.457 (95%CI: 33.992–1327.901).

**Table 2:** Meta-analysis for studies that were included in summary of ROC

Parameter	Estimate	2.5% CI	97.5% CI
Sensitivity	0.936	0.835	0.977
Specificity	0.935	0.845	0.975
False positive rate	0.065	0.025	0.155
Random effects correlation	1.000		
Θ	-0.027		
Λ	5.359		
Β	-0.027		
σ_θ_	0.000		
σ_α_	3.344		
Diagnostic odds ratio	212.457	33.992	1327.901
Likelihood ratio + ve	14.469	5.571	37.579
Likelihood ratio -ve	0.068	0.024	0.192
logit(sensitivity)	2.688	1.625	3.751
logit(specificity)	2.671	1.697	3.645
Prevalence	0.39	0.35	0.44
Accuracy	0.935

## Discussion

This meta-analysis reported an excellent pooled diagnostic value of Calprotectin in the diagnosis of PJI in comparison with gold standard methods. The pooled sensitivity and specificity of Calprotectin were 92% (Fig. 2; 95%CI: 84%-98%) and 93% (Fig. 3; 95%CI: 84%-99%), respectively. Moreover, Calprotectin was shown to provide a good diagnostic accuracy for PJI with a pooled positive likelihood ratio of 9.91 (Fig. 4; 95%CI: 4.11–23.93) and pooled negative likelihood ratio of 0.07 (Fig. 5; 95%CI: 0.02–0.22).

Eight articles with a total of 618 arthroplasties were included in this current review. High heterogeneity was reported among the included articles in terms of the type of arthroplasty (TKA, THA, TSA), diagnostic tools (ELISA, lateral flow immunoassay, immunoturbidimetric immunoassay), and cutoff point for diagnosis. Comparing individual studies, Calprotectin was found to have a better diagnostic power in the studies that included total knee replacements alone. Salari et al. reported 100% sensitivity and 95% specificity for the diagnosis of infected TKA in comparison with ICM 2018 with an AUC of 0.996. Similarly, Warren et al. compared Calprotectin to the MSIS criteria in the diagnosis of PJI in TKA; they reported high sensitivity and specificity of 98% and 96%, respectively. In addition, Grassi et al., who used the ICM 2018 criteria as a reference, reported a sensitivity and specificity of 92.3% and 100%, respectively. On the other hand, using Calprotectin in a mixed cohort of patients yielded a relatively lower diagnostic value. In the study by Wouthzyn-Bakker (2017), the diagnostic accuracy of Calprotectin was assessed in the diagnosis of PJI in 10 TKAs, 45 THAs, 5 TSAs, and 1 total ankle arthroplasty using lateral flow immunoassay with cutoff value of 50 mg/L. The authors reported 89% sensitivity and 90% specificity in comparison with the MSIS criteria. In comparison with the ICM 2018 criteria, Trotter et al. reported an AUC as low as 0.78 for Calprotectin in diagnosing PJI in TKA and THA with a sensitivity and specificity of 75%. The authors used lateral flow immunoassay with a cutoff point of 14 mg/L. As a new method used to detect PJI, however, there is no consensus about the most accurate diagnostic tool and threshold for Calprotectin. Five of the included studies used 50 mg/L as a cutoff using either ELISA (Salari, Warren), lateral flow immunoassay (Wouthuzyn-Bakker 2017 and 2018), or both methods (Grassi). Higher sensitivity and specificity were reported using ELISA with a higher PLR and a lower NLR. Grzelecki et al. used Immunoturbidimetric Calprotectin immunoassay with a threshold of 1.5 mg/L in diagnosing hip and knee PJI. They reported 95% sensitivity and specificity. This demonstrated that the method used can affect the diagnostic accuracy and lateral flow immunoassay might be inferior to other methods. Moreover, with the available literature, a threshold for diagnosis cannot be determined and more studies are needed.

Low cost, availability, and previous utilization for other pathologies are considered advantages for the use of Calprotectin in diagnosing PJI. In comparison with other available biomarkers, Calprotectin showed promising and comparable results. A pooled sensitivity and specificity of Alpha-Defensin of 95% and 96%, respectively, were reported in two recent meta-analyses [21, 22]. The meta-analysis by Wyatt et al. [23] reported pooled diagnostic sensitivity and specificity of leukocyte esterase for PJI were 0.81and 0.97, respectively. Furthermore, IL-6 showed a pooled sensitivity of 83% and a pooled specificity of 91% in the meta-analysis by Yoon et al. [24]. Future comparative controlled studies are needed to draw a solid conclusion on the value of Calprotectin in comparison with other available biomarkers in the diagnosis of PJI.

A recently published meta-analysis by Xing et al. investigated the role of Calprotectin in diagnosing PJI. However, this article did not account for the fact that the majority of the included studies used different cutoff points. Moreover, they did not specify which cutoff point was used when they conducted the analysis on the studies that reported different cutoff points. On the other hand, our study accounted for this limitation by using the Youden Index. This index guided our decision on which cutoff value to use in our analysis. Using Youden to guide us on which cutoff point to use in the analysis explains the variation in the results between our article and Xing’s meta-analysis. In addition, this meta-analysis included eight studies with a total number of 618 patients, a 15% larger sample size when compared the Xing’s meta-analysis. The larger sample size results in lower standard of error and hence lower confidence intervals across all the analyses. The new study made huge effect on the conference intervals, which is reflected on the reliability of our results.

### Limitations

Several limitations should be acknowledged in this study. First, the low number of the included studies hindered our ability to perform sensitivity analysis for different Calprotectin cutoff points or testing techniques. Second, since there is no standard technique or cutoff point for testing synovial fluid Calprotectin, different studies used different techniques and different values, which can impact the diagnostic accuracy. Accordingly, future large-scale prospective randomized trials are required to address these problems. The study by Wouthuyzen-Bakker [17] included some of the patients who were recruited in their previous study in 2017 [16], which might have created some crosspoints in our analysis. However, not all the patients were included and the sequel study included a significant number of patients. Another limitation is the fact that most of the included studies did not mention any information about blinding, sampling point time, and adjustment for confounding variables, which increases the risk of both confounding and selection biases. Finally, our analysis revealed high heterogeneity among the included studies, which can be explained by different cutoff points and Calprotectin testing techniques used by the included studies.

## Conclusion

Based on this meta-analysis, Synovial Calprotectin is a reliable and valid biomarker for PJI. It has the potential to be used in clinical practice due to its high sensitivity and specificity that are comparable to the other utilized biomarkers. Another advantage is its low cost relative to other tests. The role of Calprotectin in PJI diagnosis still needs to be elucidated in randomized trials.

## Data Availability

Not applicable.
